# A Thermally Stable Piezoresistive Textile for Reliable Tactile Sensing

**DOI:** 10.1002/advs.202511041

**Published:** 2025-09-12

**Authors:** Boxiao Li, Jianqiao Hu, Xiao Xiao, Shreesh Karjagi, Farid Manshaii, Mingchen Ma, Zhen Li, Jian Zhou, Jun Chen

**Affiliations:** ^1^ School of Material Science and Engineering Key Laboratory for Polymeric Composite & Functional Materials of Ministry of Education State Key Laboratory for Optoelectronic Materials and Technologies Guangzhou Key Laboratory of Flexible Electronic Materials and Wearable Devices Laboratory of Advanced Electronic and Fiber Materials Sun Yat‐sen University Guangzhou Guangdong 510275 China; ^2^ Department of Bioengineering University of California, Los Angeles Los Angeles CA 90095 USA

**Keywords:** piezoresistive textile, tactile sensing, ultrahigh temperature, wearable bioelectronics

## Abstract

Robotic applications in high‐temperature environments demand flexible tactile sensors that can endure extreme heat. Conventional sensors, typically made of polymers and carbon‐based materials, deteriorate quickly under such conditions. This work introduces a novel piezoresistive textile for stable tactile sensing above 495°C. The exceptional durability and heat resistance come from the robust core–shell design of the materials, featuring a silicon oxycarbide core and an amorphous carbon shell, which demonstrates unparalleled mechanical strength and flame resistance. With airflow‐assisted rotary spinning and density‐controlled sintering, the piezoresistive textile is scalable and consistently performs at temperatures up to 250°C for over 24 h and can withstand even higher temperatures of 495°C for 4 h. When integrated into a robotic gripper, the ultrahigh temperature textile sensor successfully retrieves a small item from flames fueled by alcohol, showcasing its effectiveness in high‐temperature tactile sensing. This innovative textile sensor offers a promising solution for tactile sensing and robotic applications in high‐temperature environments.

## Graphical abstract

A heat‐resistant piezoresistive textile based on silicon oxycarbide/carbon core–shell fibers is developed using scalable airflow‐assisted spinning. The textile retains tactile sensing performance at 250°C for 24 h and endures temperatures up to 495°C for 4 h. When integrated into a robotic gripper, it enabled object retrieval from open flames, demonstrating its potential for extreme‐environment robotics.

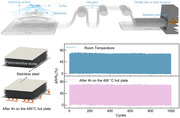

## 1 Introduction

Flexible tactile sensing plays a vital role in advancing robotic systems, enabling seamless human‐robot interaction, and supporting machine learning applications.^[^ ^1^, ^2^, ^3^, ^4^, ^5^, ^6^ ^]^ Beyond replicating human touch, this technology empowers robots to operate and adapt in extreme environments that are often inaccessible to humans. For instance, robotic systems deployed near volcanic craters (≈ 1000°C) or engaged in fire rescue missions (up to 500°C) rely on functional sensors to successfully detect hazards and carry out life‐saving operations. Flexible tactile sensors often rely on elastic polymers as substrates or matrix materials. Many studies have explored coating polydimethylsiloxane substrates with conductive materials like metals or carbon.^[^ ^7^, ^8^ ^]^ Substrate deformation induces crack formation in the conductive layer, increasing electrical resistance and manifesting the piezoresistive effect.^[^ ^9^, ^10^, ^11^, ^12^, ^13^, ^14^ ^]^ However, these sensors have limited durability at extreme temperatures.^[^ ^15^, ^16^, ^17^ ^]^ Most polymers stiffen at low temperatures and degrade at high temperatures. Research has also explored alternative sensor designs that do not rely on polymeric substrates.^[^ ^18^ ^]^ Recent studies reported lamellar graphene aerogels with exceptional piezoresistive sensitivity for monitoring human body strain signals.^[^ ^19^ ^]^ Carbon nanofiber aerogels derived from bacterial cellulose demonstrated excellent thermal insulation and piezoresistive response.^[^ ^20^ ^]^ While carbon‐based materials can withstand low temperatures, they are susceptible to rapid oxidation, especially at elevated temperatures and in oxygen‐rich environments.^[^ ^21^ ^]^

High‐temperature environments pose significant challenges for tactile sensors, as conventional materials degrade rapidly under extreme heat. To tackle this issue, our research introduces a novel ultrahigh temperature textile (UTT) sensor, developed using airflow‐assisted rotary spinning (AARS) technology, which offers remarkable durability and scalability. The sensor features a core–shell structure, consisting of an amorphous carbon shell surrounding a silicon oxycarbide core, enabling it to maintain stable performance at temperatures up to 250°C for over 24 h and withstand a butane flame for 3 min. When integrated into a robotic gripper, this sensor provides advanced tactile sensing capabilities, allowing the system to perform tasks in extreme conditions and successfully retrieve items, such as USB drives from flames. This innovation demonstrates significant potential for critical applications, including data protection in harsh environments, overcoming longstanding material limitations and advancing robotics for extreme settings.

## 2 Results and Discussion

**Figure 1:**
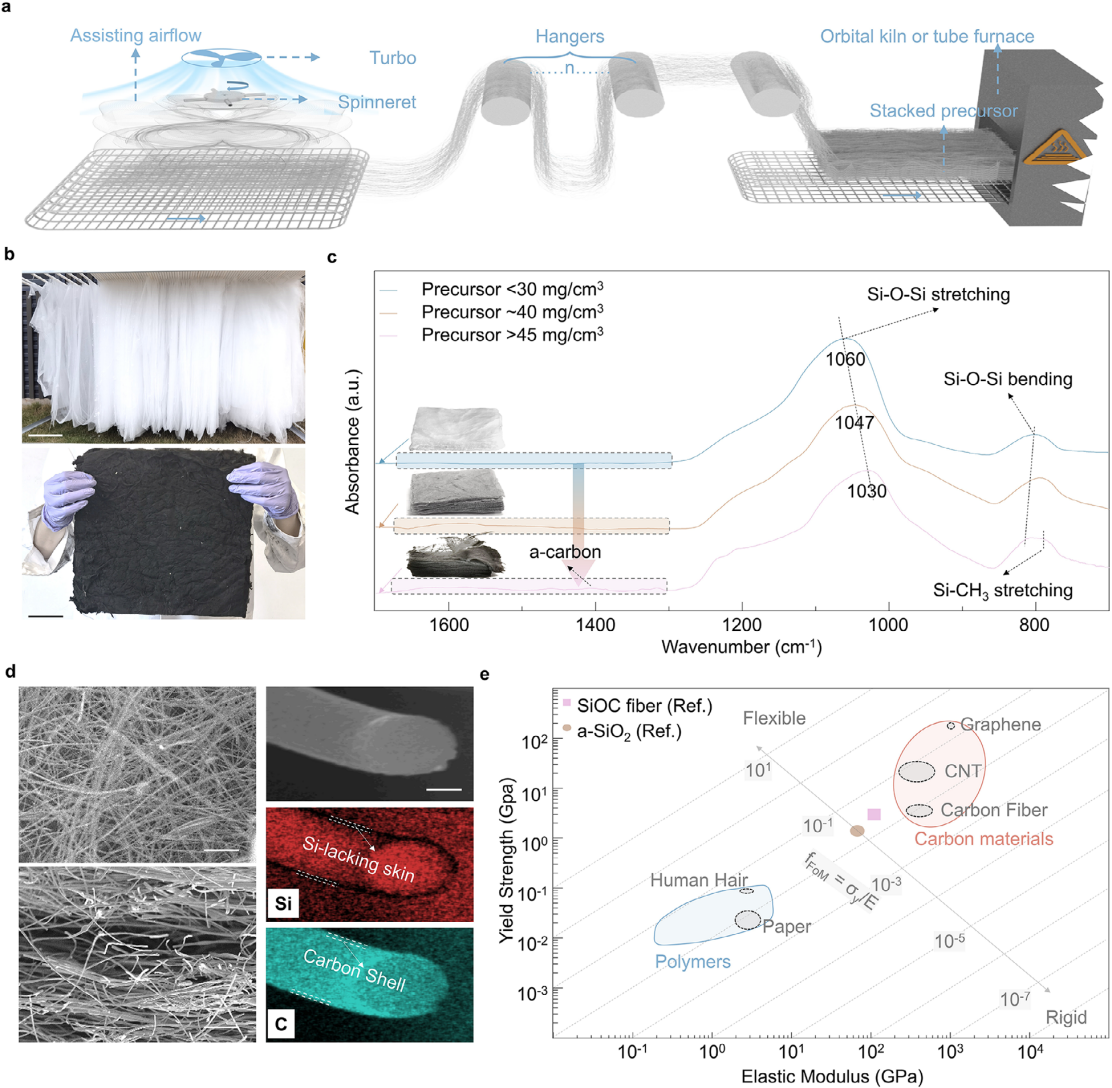
The Fabrication Process and Characteristics of Piezoresistive Textiles. a) Schematic of the airflow‐assisted rotary spinning technology. b) Photograph of the as‐spun precursor on the hangers (top, scale bar: 15 cm) and carbonized piezoresistive textile (bottom, scale bar: 4 cm). c) FTIR spectra of calcinated precursors at various packing densities. d) SEM images showing the piezoresistive textile and element distributions within the core–shell piezoresistive fiber (scale bar: 20 µm). e) Comparison of the figure of merit for flexibility (FoMf) between SiOC and other materials. Reproduced with permissions.^[^ ^34^ ^]^ Copyright 2019, American Association for the Advancement of Science.

The UTT, with its core–shell design, demonstrates unparalleled mechanical strength and flame resistance.^[^ ^22^, ^23^ ^]^ Among various spinning methods, electrospinning enables sub‐micron fiber production. Still, it has a low yield of 5–10 mL h^−1^,^[^ ^24^, ^25^ ^]^ while centrifugal spinning offers higher yields but achieving directed fiber collection is challenging due to the divergent nature of the centrifugal force.^[^ ^26^ ^]^ To address these limitations, we developed an AARS technique (**Figure** **1**a). This technique combines centrifugal force with controlled airflow directed perpendicular to the conveyor belt. As the polymer solution exits the rotating spinneret, the centrifugal force stretches the material. Concurrently, airflow guides the fibers onto the moving conveyor belt, enabling the deposition of layered, porous textiles with good mechanical continuity.

For large‐scale production, we employed a system with eight spinning heads, each containing eight needles, positioned above a conveyor belt (Figure 1b, top). After achieving a steady state, the precursor material (a polyvinylpyrrolidone (PVP) template and tetraethylorthosilicate (TEOS) silica precursor) was collected and transferred for further processing. A 60 min cycle yielded a 3 kg fiber mat draped over the collector. The as‐spun precursor undergoes carbonization in a furnace or kiln. The final product's color depends on the density of the layered precursor during this process. Densities below 0.03 g cm^−^^3^ result in white samples, while those between 0.03 and 0.04 g cm^−^^3^ turn gray. Black samples, exhibiting the desired conductive properties, are achieved at densities between 0.04 and 0.06 g cm^−^^3^ (Figure 1c).

Fourier Transform Infrared (FTIR) analysis confirms the presence of PVP in the precursor, identified by characteristic absorption bands at 1637 and 1279 cm^−1^, corresponding to the C═O stretching in the pyrrolidone group and the C─N bending vibration, respectively (Figure S1, Supporting Information).^[^ ^27^, ^28^ ^]^ For the conductive samples, the spectrum exhibits absorption bands indicative of Si─C and Si─O─Si bonds, with bands centered at 795, 810, 1030, and 1235 cm^−1^, associated with Si─C bending, Si─O─Si bending, Si─O─Si stretching, and Si─C stretching, respectively.^[^ ^29^, ^30^ ^]^ Compared to the white sample (typical SiO_2_ IR spectrum), the sample exhibits blue shifts in the Si─O─Si bending and redshifts in Si─O─Si stretching, attributable to changes in the Si─O─Si bond angle due to the presence of Si─C bonds. Additionally, absorption bands between 1300 and 1700 cm^−1^ suggest the presence of amorphous carbon_._^[^ ^31^ ^]^ These results confirm the core–shell structure of the fibers: an amorphous carbon outer shell (C) and a SiOC core.

The presence of the amorphous carbon layer leads to anisotropic electrical conductivity. Measurements taken along the stack direction (σ_┴_) and parallel to the stack (σ_ǁ_) show significantly different values (0.026 and 0.8 S m^−1^, respectively). This reflects the influence of the material's structure on its electrical properties. The scanning electron microscopy (SEM) image in Figure 1d illustrates this anisotropy. The top‐view image shows continuous fibers within each layer, facilitating in‐plane electron transfer. The cross‐section reveals vertical connections formed by intersecting cylindrical fibers, creating narrow interfaces with higher resistance. Energy‐dispersive X‐ray spectroscopy (EDS) analysis confirms a uniform distribution of silicon (red) throughout the material, except for the edge of the cross‐section, indicating a silicon‐rich core (Figure 1d). SEM analysis reveals that the fiber core has a diameter of 840 nm, encased by a ≈ 50 nm thick carbon shell. The absence of silicon on the fiber surface, together with the carbon distribution (shown in blue) matching the fiber diameter in SEM images and supported by FTIR analysis, confirms the core–shell structure. The structure features a silicon oxycarbide core encased in an amorphous carbon shell. The total fiber diameter ranges from 0.6 to 2.2 µm, with the carbon shell comprising 8% to 12% of the overall diameter. Due to the carbon shell, the layered textile mat (Figure S2a, Supporting Information) exhibits hydrophobic feature with water contact angle of 137.2° (Figure S2b, Supporting Information). This high WCA of the textile provides the potential for resistance to humidity.^[^ ^32^, ^33^ ^]^ In addition, the uniformity of the textile is assessed through EDS images at various magnifications (Figure S2c–e, Supporting Information), confirming the consistent distribution of carbon. The flexibility of a material within its elastic range depends on its geometric dimensions (thickness) and its figure of merit for flexibility (FoM_f_), as described by Equation (1).^[^ ^34^ ^]^

(1) $$f\;=\frac{1}{r_{b}}=\left(\frac{2}{h}\right)\;\times \;\left(\frac{\sigma_{y}}{E}\right)=\left(\frac{2}{h}\right)\;\times FoM_{f}$$

where f, h, σ_y_, E are flexibility, thickness, yield strength, and Young's modulus of the material, respectively. As shown in Figure 1e, the SiOC material has a FoM_f_ value of 0.05, comparable to that of carbon materials and some polymeric substances like human hair and paper. Additionally, the average fiber diameter is 1.2 ± 0.3 µm (Figure S3, Supporting Information), suggesting that this textile is likely to exhibit good flexibility and can tolerate significant bending curvature within its elastic range.

**Figure 2:**
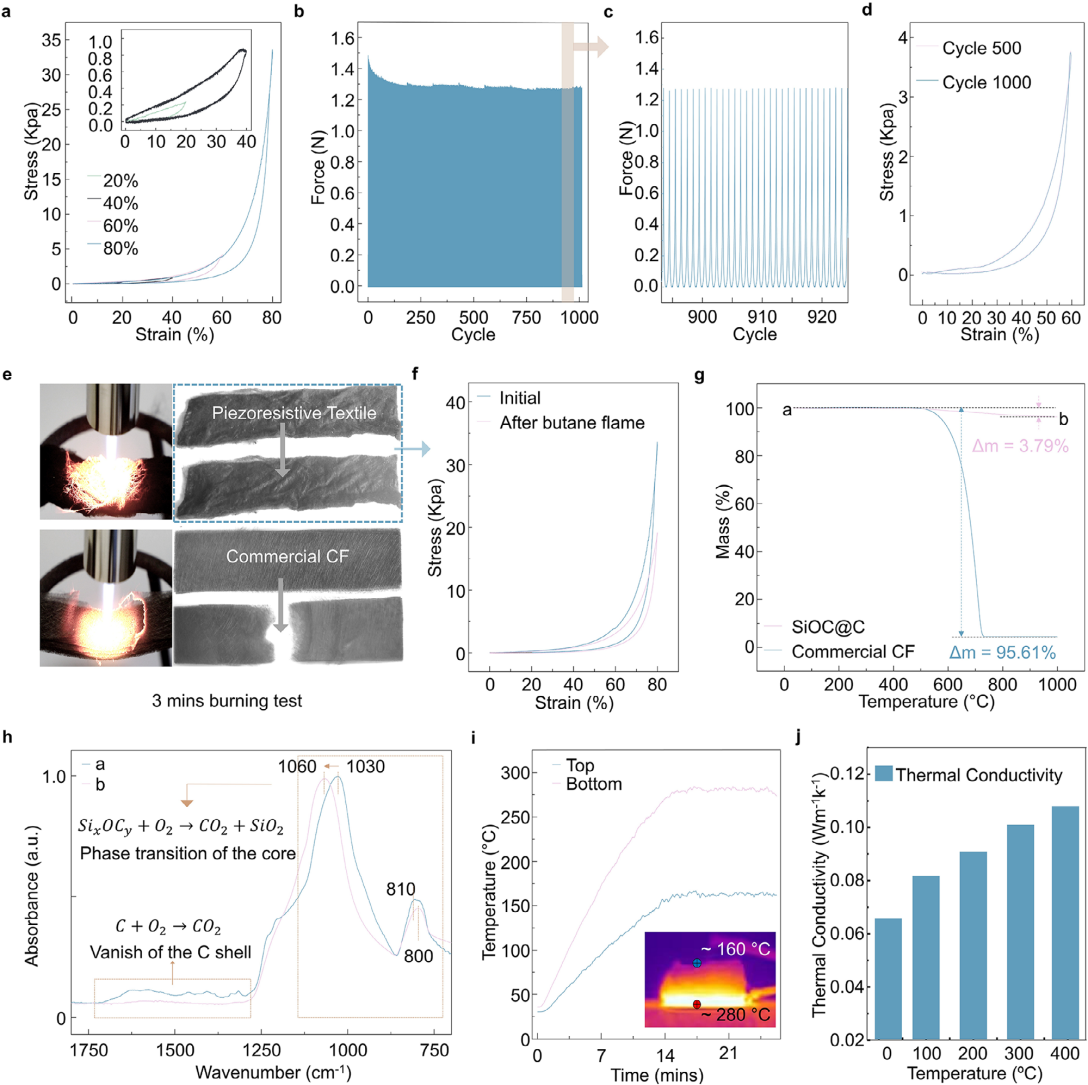
Mechanical Performance and Flame Resistance of the Piezoresistive Textile. a) Compressive stress–strain curves at 20%, 40%, 60%, and 80% of the textile. b) Durability test for up to 1000 cycles at 60% strain. c) Detailed view of the selected cycles. d) Stress–strain curves of the piezoresistive textile at the 500th and 1000th cycles. e) Flame retardant comparison between piezoresistive textile and commercial carbon fiber (CF). f) Stress–strain curves before and after exposure to a butane flame. g) Thermogravimetric analysis of the commercial CF and piezoresistive textile. h) FTIR spectra of the piezoresistive textile before and after TGA. i) Thermal conductivity results for the stacked piezoresistive textile. j) Thermal conductivity of the sensor with different temperatures.

To evaluate the mechanical properties, compressive stress testing was performed on the textile with a gauge length of 1 cm. As demonstrated in **Figure** **2**a, the textile exhibits remarkable resilience, capable of rebounding from compressive strains of 20%, 40%, 60%, and even 80%. Notably, at an 80% strain, the stress reaches 32 kPa. Photographic evidence of this resiliency is presented in Figure S4 (Supporting Information). Furthermore, the textile demonstrates exceptional mechanical durability, retaining 86.2% of its initial stress after 1000 compressive cycles (Figure 2b). This exceptional stress retention highlights the material's robustness, making it suitable for long‐term applications. Detailed cyclic test results in Figure 2c reveal that the maximum compressive stress remains stable without significant fluctuations. Moreover, the stress–strain curves at 500 and 1000 cycles nearly overlapped, confirming the textile's exceptional mechanical resilience (Figure 2d). These findings demonstrate that the textile possesses a unique combination of high compressibility, resilience, and durability, making it a promising candidate for applications requiring materials that can withstand repeated mechanical stresses without significant degradation in performance.

As shown in Figure 2e, the textile retains its morphology after a 3 min exposure to a butane flame, unlike commercial carbon fiber (CF) nonwovens, which suffered structural degradation due to oxidation under the same conditions. Even after exposure, the post‐exposure compressive stress–strain curve (Figure 2f) demonstrates that the textile maintains resilient behavior, with a maximum compressive stress of 20 kPa. This demonstrates the material's ability to preserve its mechanical integrity even after exposure to high temperatures. Thermogravimetric analysis (TGA) and FTIR were employed to further investigate the thermal stability of the textile (Figures 2g,h). The combined results show the superior thermal stability of the textile, with a significantly lower mass loss of 3.79% compared to 95.61% for the commercial CF textile when exposed to temperatures up to 1000°C. In addition to its reliable mechanical properties, the textile's high‐temperature resistance is crucial for enabling piezoresistive sensing in extreme environments. As the temperature increases from 20 to 250°C, the textile's electrical resistance decreases significantly, from 5 kΩ to 1.6 KΩ (Figure S5a, Supporting Information). Following prolonged exposure to 250°C and subsequent cooling to room‐temperature, the resistance stabilizes at 9.8 kΩ within 1 h. In contrast, exposure to 495°C for several hours results in a significant increase in resistance, reaching ≈ 490 kΩ after 4 h (Figure S5b, Supporting Information). This increase is likely due to oxidation of the carbon shell. While thermogravimetric analysis (TGA) shows an oxidation onset above 500°C, slow oxidation can still occur at lower temperatures. Despite the significant rise in resistance, the textile maintains its piezoresistive behavior even after 4 h of exposure (Figure S5c, Supporting Information). Furthermore, the hysteresis remains comparable in magnitude before and after the high‐temperature treatment, with a hysteresis resistance of ≈ 8%. The textile's pressure sensitivity (S) is measured at 5.4 kPa^−1^ (Figure S5d, Supporting Information), comparable with the previously reported textile sensors.^[^ ^35^ ^]^ The FTIR spectrum at point “b” suggests that as the temperature increases, the amorphous carbon shell oxidizes, while the fiber core transitions from SiOC to SiO_2_. Given its low density of 58 mg cm^−3^, comparable to many aerogel materials, the thermal conductivity of the textile was investigated. As shown in Figure 2i, a 9 mm thick stacked textile placed on a hot plate heated to 280°C registers a surface temperature of 160°C. The thermal conductivity (κ) of the textile increases from 0.065 W m^−1^ k^−1^ at 20°C to 0.109 W m^−1^ k^−1^ at 400°C (Figure 2k). Remarkably, even after 3 min exposure to the butane flame (Figure 2e), the textile retains 80% of its initial parallel conductance. This indicates that despite surface oxidation of the amorphous carbon, its low thermal conductivity acts as a barrier, preventing further oxidation and enabling self‐protection. Furthermore, the high porosity of the piezoresistive textile facilitates enhanced permeability (Note S1, Supporting Information).

**Figure 3:**
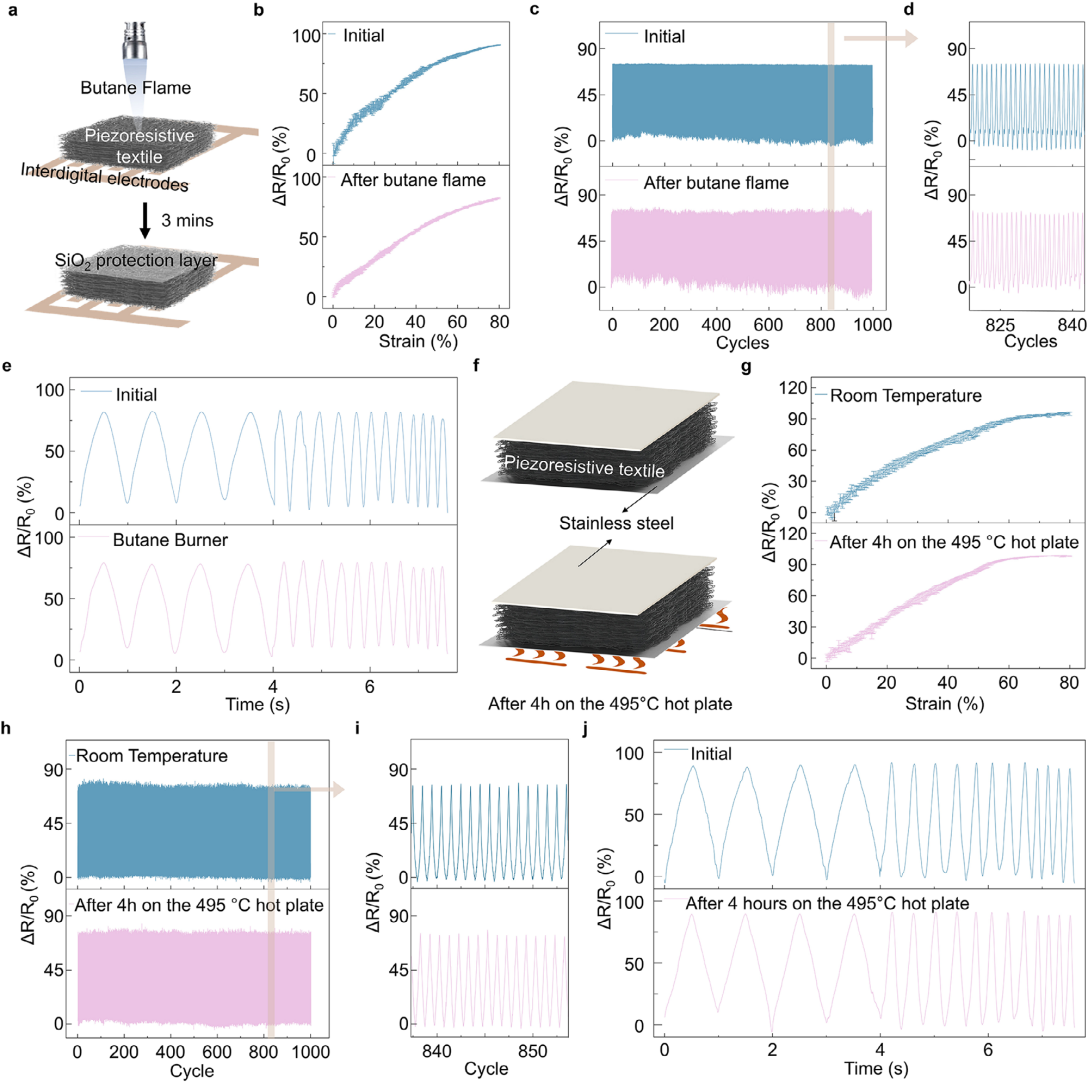
Tactile Sensing Performance of the Piezoresistive Textile Sensors. a–e) Piezoresistive performance of the UTT sensor with interdigital electrodes. f–j) Piezoresistive performance of the UTT sensor with double‐sided electrodes. Schematic of the sensor and butane flame spray (a). Piezoresistive response of the sensor (b,g). Piezoresistive cycling test with strain from 20% to 60%. (c,h). Zoomed‐in piezoresistive cycling test (d,i). The response of the sensor to different frequencies (1, 3, 4, and 5 Hz) within a strain range of 20–60%, with four cycles conducted at each frequency. (e,g). Schematic of the sensor and the hot plate test (f).

To evaluate the UTT sensor's functionality and high‐temperature resistance, a sensor with interdigital electrodes was fabricated and tested (**Figure** **3**a). The UTT sensor exhibits a piezoresistive response up to 80% compressive strain, with a linear gauge factor (GF) of 1.3 within the initial 60% strain (Figure 3b). Even after exposure to a butane flame for 3 min, the GF remains at 1.2, indicating minimal impact on performance. This is likely due to the formation of a protective silicon dioxide layer upon exposure to the flame and the material's inherently low thermal conductivity, which prevents significant internal temperature increase and oxidation. This phase transition is confirmed by FTIR analysis of the textile's top layers (Figure S6, Supporting Information). Consequently, the sensor retains its piezoresistive functionality. Furthermore, the UTT sensor demonstrates promising cycling stability within a compressive strain range of 20–60% (Figure 3c,d). The resistance change rate varies from 0% (decompressed) to 72% (compressed) and remains stable over 1000 cycles. Even after flame exposure, it exhibits a similar variation, ranging from 0% to 68% in the compressed state after 1000 cycles. The UTT sensor exhibits a frequency response range from 1 to 5 Hz for compress‐decompress cycles, both before and after the 3 min flame exposure (Figure 3e). This highlights the material's robust performance and stability under thermal stress, making it a promising candidate for high‐temperature piezoresistive sensing applications.

It is important to note that sensors assembled with stainless steel electrodes on both top and bottom faces are not suitable for high‐temperature applications due to electrode deformation at elevated temperatures. To further assess the UTT sensor's high‐temperature performance, a sensor was placed on a hot plate at 495°C for 4 h (Figure 3f). Infrared imaging confirmed that the bottom side reached 495°C, while the top side attained 200°C (Figure S7, Supporting Information). Similar to the sensor with interdigital electrodes, this sensor maintains a linear piezoresistive response from 0% to 60% strain, with a GF of 1.50 before heating and 1.52 after heating (Figure 3g). Moreover, the UTT sensor displays stable cycling performance, with a nearly constant resistance change observed throughout 1000 compress–decompress cycles, even after exposure to the hot plate (Figures 3h,i). The frequency response of this sensor (Figure 3j) is similar to the one with interdigital electrodes, effectively handling operations with high‐frequency dynamics up to 5 Hz, demonstrating its suitability for high‐temperature environments.

**Figure 4:**
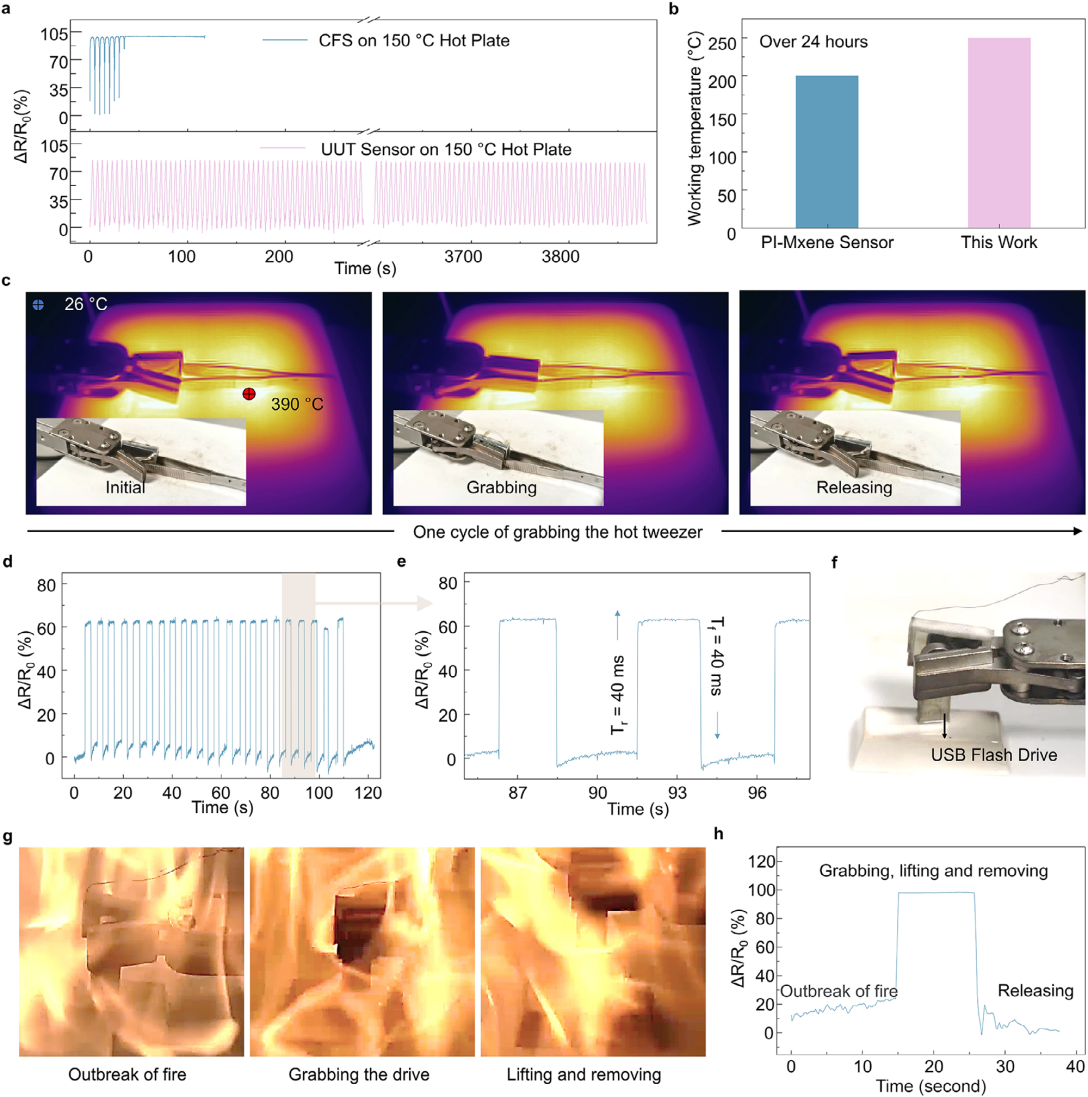
Demonstration of the Tactile Sensing Capabilities in the Machine Gripper at high‐temperature. a) Comparison of piezoresistive performance between a carbon‐based flexible sensor and a UTT sensor. b) Temperature endurance comparison over 24 h between MXene‐based sensors and the UTT sensors. c) Infrared and corresponding visual images of the gripper grasping a tweezer. d) Cycling tactile sensing performance of the gripper. e) Detailed view of the piezoresistive results during cycling tests. f) Photograph of the gripper preparing to retrieve a USB drive. g) Photographs of the gripper rescuing the USB flash drive from fire. h) Tactile sensing performance of the gripper during the rescue process.

To thoroughly assess the UTT sensor's high‐temperature resistance, a comparative analysis was conducted between the UTT sensor and a commercially available flexible carbon‐based sensor (Resistive pressure film sensor, RX‐D2027). As shown in **Figure** **4**a, during tests on a hot stage set at 150°C, the commercially available sensor maintained functionality for only 30 s. In contrast, the UTT sensor with double‐sided electrodes operated uninterruptedly for an impressive 280 s, even after being subjected to the 150°C hot stage for 1 h. Furthermore, the UTT sensor exhibited long‐term durability, maintaining consistent piezoresistive functionality after exposure to 250°C for over 24 h and in an oven 495°C for over 4 h in a furnace, as confirmed by subsequent piezoresistive cycling tests (Figure S8, Supporting Information). The stability of the UTT sensor stems not only from the consistent piezoresistive hysteresis behavior of the textile but also from its preserved structural integrity after high‐temperature exposure. Even after 4 h at 495°C, the stress–strain curve remains nearly unchanged from its initial state (Figure S9a, Supporting Information). Additionally, 1000 cycles of strain–stress testing (0%–60% strain) show that the textile's mechanical response stabilizes after the first 100 cycles and remains consistent thereafter (Figure S9b, Supporting Information). However, prolonged exposure to 495°C in a furnace for 5 h leads to unstable piezoresistive cycling due to gradual surface oxidation, which causes an uneven distribution of the conductive carbon shell (Figure S10, Supporting Information). Moreover, as shown in Figure 4b, the UTT sensor outperformed previously reported polyimide and MXene‐based sensors,^[^ ^36^ ^]^ which withstand 200°C for more than 24 h. Additional comparisons of temperature and duration are presented in Table S1 (Supporting Information), further highlighting the textile's excellent thermal resistance.

To demonstrate the practical application of the piezoresistive textile, a machine gripper equipped with a UTT sensor was developed to enhance its tactile sensing capabilities for high‐temperature environments Figure 4c presents one cycle of the gripper engaging a tweezer on the hotplate. Figure 4d illustrates the piezoresistive response of the gripper across 22 cycles of grabbing and releasing objects at high temperatures. The stable resistance change confirms the gripper's capability to securely handle objects. The UTT sensor's rapid response is evident from the 40 ms rise time (T_r_) and fall time (T_f_) during the grabbing and releasing process (Figure 4e). The gripper's functionality was further tested in a fire scenario (Figure 4f). A USB flash drive was placed over burning alcohol fuel. The gripper successfully retrieved the USB flash drive from the fire, preserving the data within (Figure 4g). The tactile sensing response of the gripper during this operation (Figure 4h) indicates that the USB drive was exposed to fire for 15 s before being secured. Within 15 s of the fire outbreak, the temperature of the flash drive could reach a peak of 155°C, according to infrared thermal images from a parallel experiment (Figure S11, Supporting Information). The removal process lasted 10 s, after which the drive was safely released. Remarkably, the flash drive remained operational, highlighting the gripper's effectiveness in high‐temperature environments. This capability opens up potential applications in fire rescue, aerospace surveillance, and high‐temperature tactile sensing for advanced robotic systems.

## 3 Conclusion

This work presents the successful synthesis of a novel piezoresistive textile composed of core–shell fibers, fabricated using the innovative airflow‐assisted rotary spinning method. This high‐yield, precise technique enables the large‐scale production of textiles with exceptional mechanical properties and remarkable high‐temperature resilience. Notably, the piezoresistive textiles retain functional stability after exposure to severe thermal challenges, such as a butane flame for 3 min or a 495°C hot plate for 4 h. The UTT sensors also exhibit a reliable frequency response of up to 5 Hz. These properties make the piezoresistive textile a strong candidate for applications in harsh environments, particularly for robotic systems requiring dependable sensory feedback. The successful integration of the piezoresistive textile into UTT sensors for a robotic gripper demonstrates their potential to significantly improve the performance and safety of robots operating under extreme conditions. This contribution paves the way for advancements in smart textiles and high‐performance sensors, potentially transforming future robotics and industrial applications.

## 4 Experimental Section

### Fabrication of the Piezoresistive Textile

The spinning solution was prepared by mixing 6 g of tetraethylorthosilicate (TEOS, 99%, Aladdin), 17 g of polyvinylpyrrolidone (PVP, Mw = 800 000, Aladdin), and 0.04 g of phosphoric acid (PA, 99%, Aladdin) with 17 mL of deionized water. The mixture was stirred for 2 h to ensure uniformity before being fed into the spinning spinneret. The spinning apparatus operated at a rotational speed of 6000 Rad min^−1^ to form fiber precursors, while an industrial turbine with a rotation speed of 2k RPM (above the rotating spinneret), directed additional airflow (7800 m^3^ h^−1^) toward a mesh‐like conveyor belt for fiber collection. The precursor fibers were then stacked to achieve a density of 0.04 to 0.06 g cm^−3^ and carbonized in either a tube furnace or an orbital kiln. The carbonization process entailed heating at a rate of 5–10°C min^−1^ to 1000°C, maintaining this temperature for 1 h, and then cooling to room‐temperature.

### Fabrication of the Ultrahigh Temperature Textile (UTT) Sensors

For the sensor with interdigital electrodes, patterned stainless steel foils were adhered to high‐temperature‐resistant tape (3 M 8951) to create interdigitated electrodes. A 3 mm thick UTT was then positioned on top of the electrodes to assemble the sensor. For the sensor with double‐sided electrodes, a similar construction was used, involving interdigitated stainless‐steel electrodes on two substrates of high‐temperature‐resistant tape with the piezoresistive textile sandwiched in between.

### Sensor Testing

The compression, tensile, and cyclic tests of the textile were conducted using an Instron universal testing machine, with compression and release strain rates of 4% per second. During these phases, the resistance changes were monitored using a digital multimeter (KEYSIGNT, EDU34450A). Piezoresistive cycling tests were performed with a compress‐release cycle frequency of 1 Hz.

### Comparisons Between the Commercial and UTT Sensor

For comparison, the commercially available flexible piezoresistive sensor RX‐D2027 (thickness of 0.3 mm, actuation force of 0.1N) was tested under similar conditions. High‐temperature compression tests were conducted using an Instron machine and a hot plate set at 150°C. The cyclic compression test for both the UTT and commercial sensors was carried out at a speed of 100 mm min^−1^. Sensing area of the commercial sensor is 12.57 cm^2^. Resistance measurements were taken by a commercial multimeter at 100 SPS (samples per second).

### Material Characteristics

Scanning electron microscope (SEM) images and energy‐dispersive spectra (EDS) were captured using the ZEISS GeminiSEM 500. The FTIR spectrum of the textile was recorded using the Bruker Vertex70 FTIR spectrometer with an Attenuated Total Reflection (ATR) technique at a resolution of 2 cm^−1^. Thermal conductivities of the textile were measured with the Hot Disk TPS3500, and thermogravimetric analysis (TGA) was performed using the NETZSCH TG209 F1 instrument, heating from 100 to 1000°C at a rate of 10°C min^−1^ under ambient conditions.

## Conflict of Interest

The authors declare no conflict of interest.

## Supporting information



Supporting Information

## Data Availability

The data that support the findings of this study are available from the corresponding author upon reasonable request.
