# Supplementary material for: A Thermally Stable Piezoresistive Textile for Reliable Tactile Sensing
Source: Adv Sci (Weinh). 2025 Sep 12;12(42):e11041. doi: 10.1002/advs.202511041 (PMC12622492; doi:10.1002/advs.202511041)
Supplement: Supplementary file 1 — Supporting Information [file ADVS-12-e11041-s001.docx]

Supporting Information

A Thermally Stable Piezoresistive Textile for Reliable Tactile Sensing

Boxiao Li, Jianqiao Hu, Xiao Xiao, Shreesh Karjagi, Farid Manshaii, Mingchen Ma, Zhen Li, Jian Zhou^*^, Jun Chen^*^

**This Supporting Information includes：**

**Figure S1**: ATR-FTIR of PVP-templated Precursor.

**Figure S2**: Layer-by-Layer Structure, Surface Wettability and uniformity of the Textile.

**Figure S3**: Diameter Distribution of the Core-shell Piezoresistive Fibers.

**Figure S4**: Photographic Evidence of the Textile's Resiliency During the Test.

**Figure S5**: Electrical Resistance Characteristics of the Piezoresistive Textile.

**Figure S6**: FTIR Spectrum of the Top Layers of the Textile After Exposure to the Butane Flame.

**Figure S7**: Durability test for the sensor.

**Figure S8**: Infrared Image of a 3 mm Thick UTT Sensor on a 495°C Hot Plate.

**Figure S9**: Mechanical Resilience of the Textile.

**Figure S10**: Surface Morphology of Fiber for Failure Analysis.

**Figure S11**: Infrared Imaging of a USB Flash Drive in an Alcohol Flame for 15s.

**Table S1**: Comparison of Thermal Stability with Other Flexible Piezoresistive Sensors.

**Note S1**: Permeability assessment of the piezoresistive textiles.


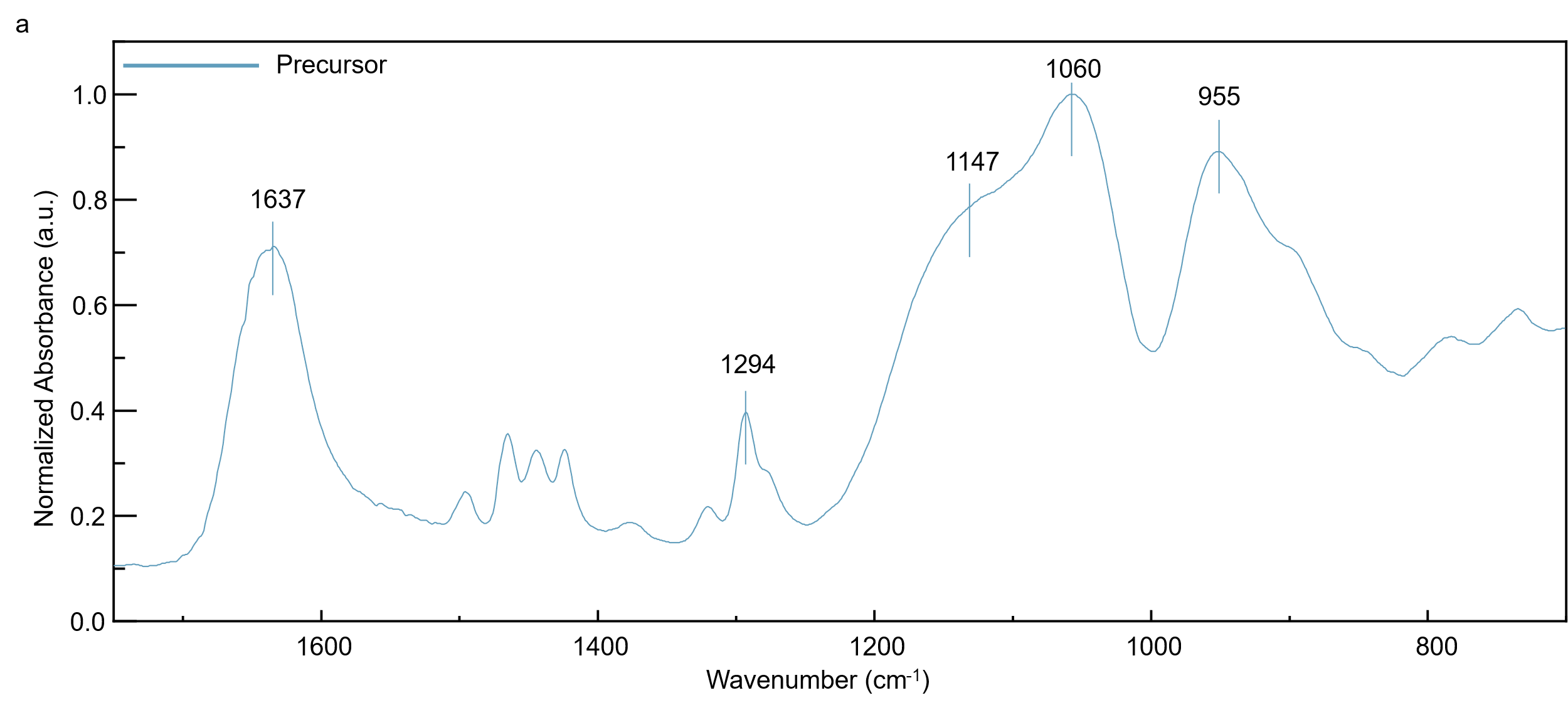


**Figure S1.** ATR-FTIR of PVP-templated Precursor.

For the FTIR spectra of the PVP-based precursor (**Figure S1**), the absorption bands located around 1637 cm^-1^ and 1279 cm^-1^ are associated with PVP, referring to the stretching of C=O in the pyrrolidone group and the C–N bending vibration of pyrrolidone, respectively.^[1, 2]^ In addition, the bands at 1147, 1060, and 955 cm^-1^ are related to TEOS and can be attributed to Si-O-Si asymmetry stretching, Si-O-Si symmetry stretching, and rocking of C-H respectively.^[3]^


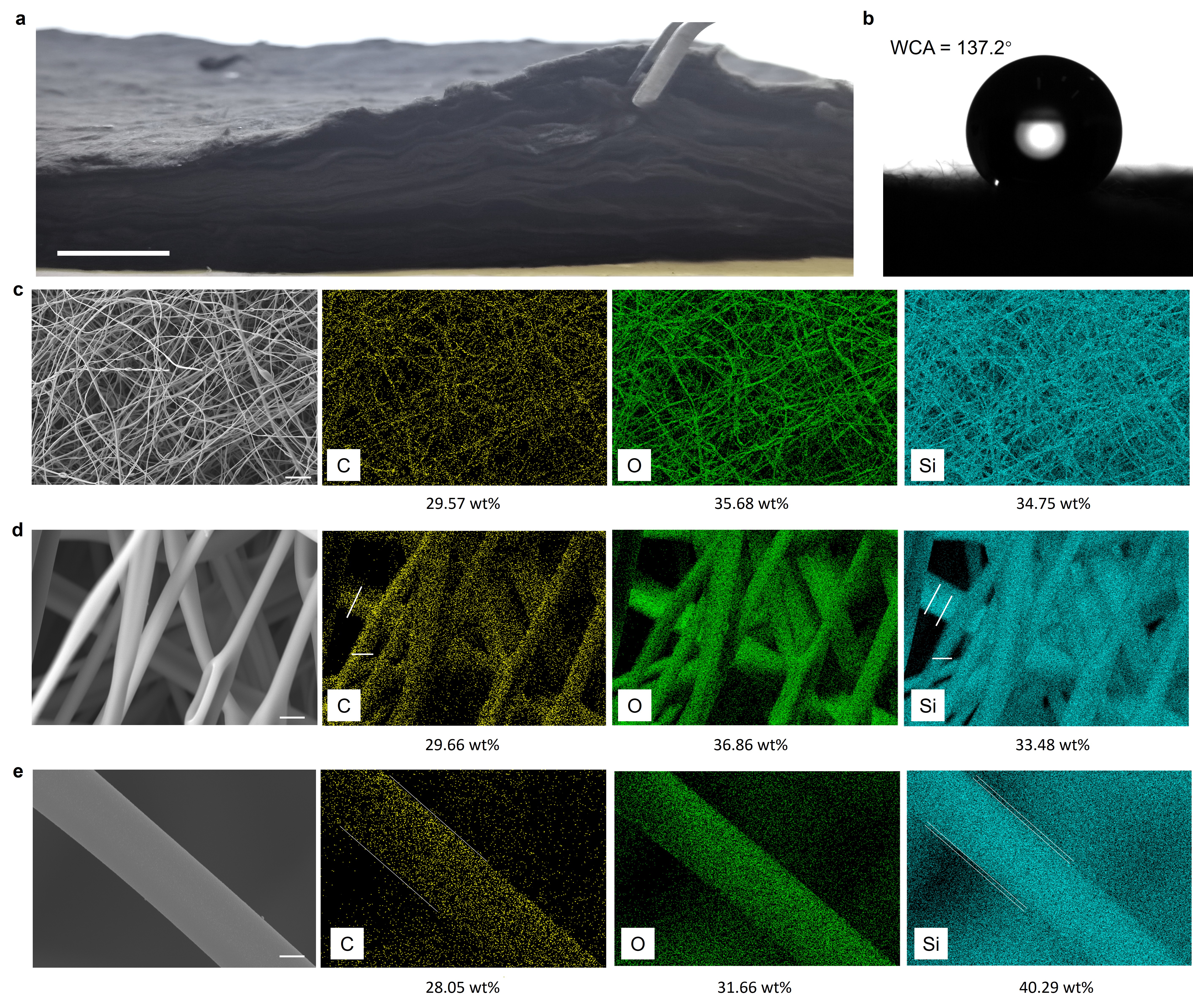


**Figure S2. Layer-by-Layer Structure, Surface Wettability and uniformity of the Textile. a**, Photograph of the textile with layer-by layer structure (Scale bar, 1 cm). **b**, WCA of the textile. **c**–**e,** SEM and corresponding EDS images of the textile at magnifications of 200× (**c**, scale bar: 50 μm), 3,000× (**d**, scale bar: 2 μm), and 10,000× (**e**, scale bar: 500 nm).

**
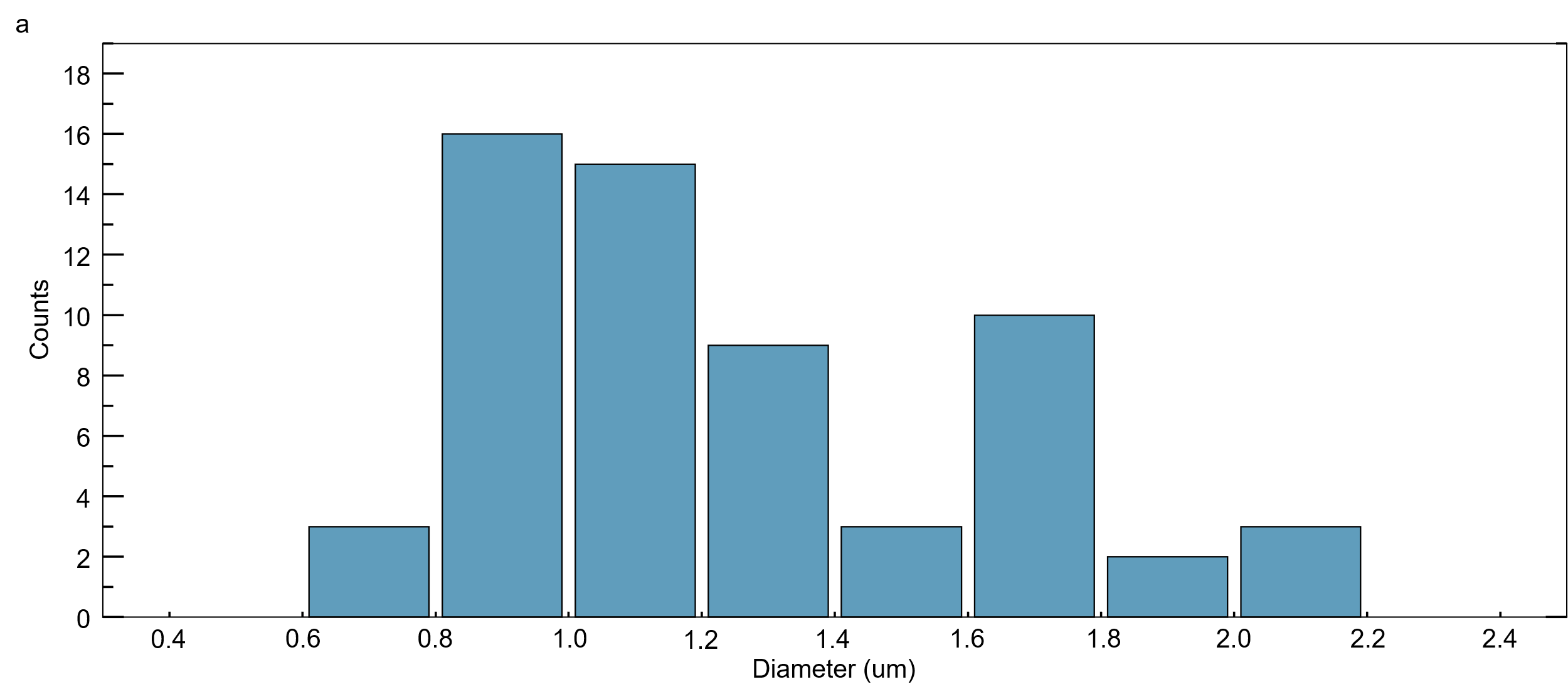
**

**Figure S3.** Diameter Distribution of the Core-shell Piezoresistive Fibers.

**
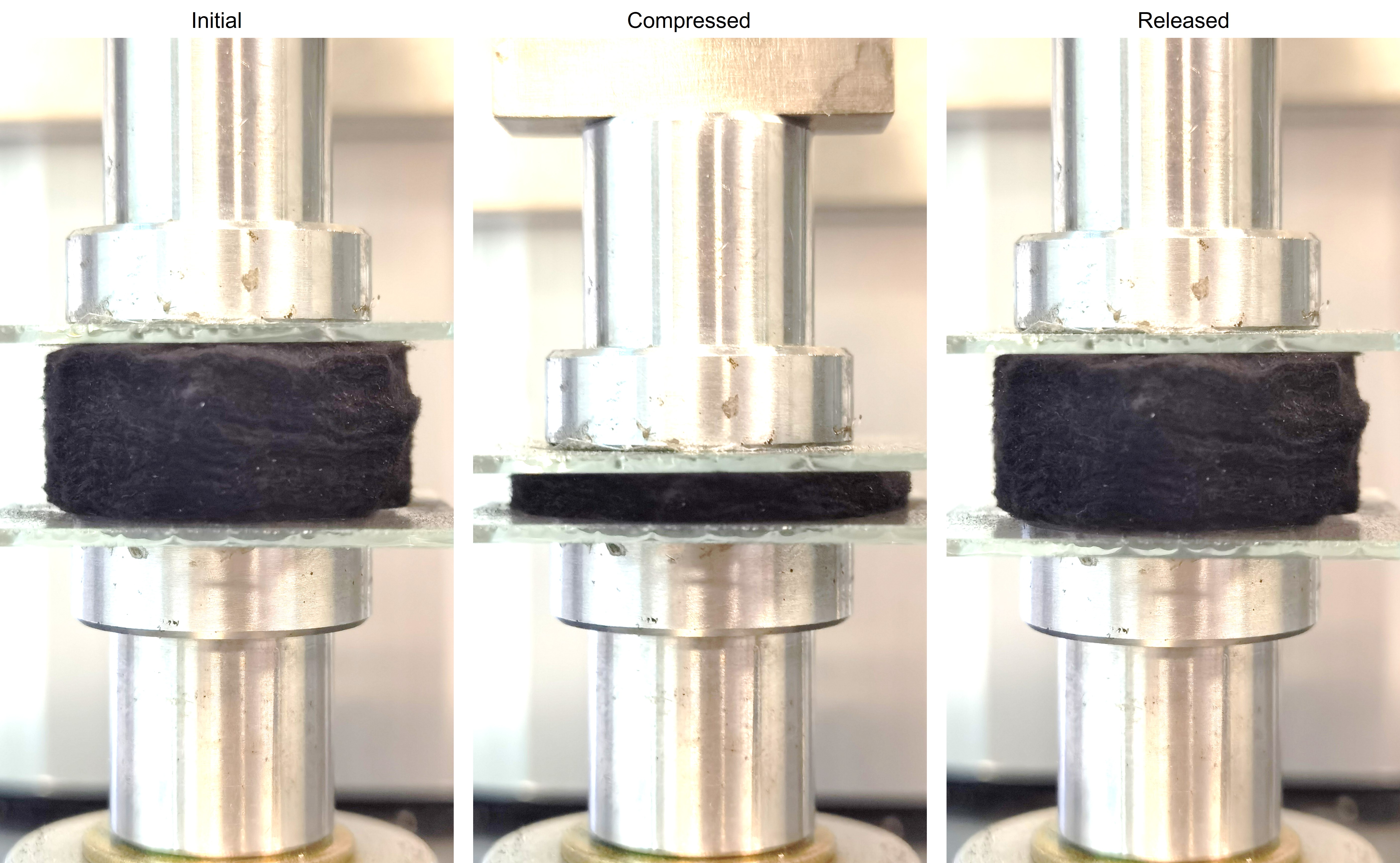
**

**Figure S4.** Photographic Evidence of the Textile's Resiliency During the Test.

**
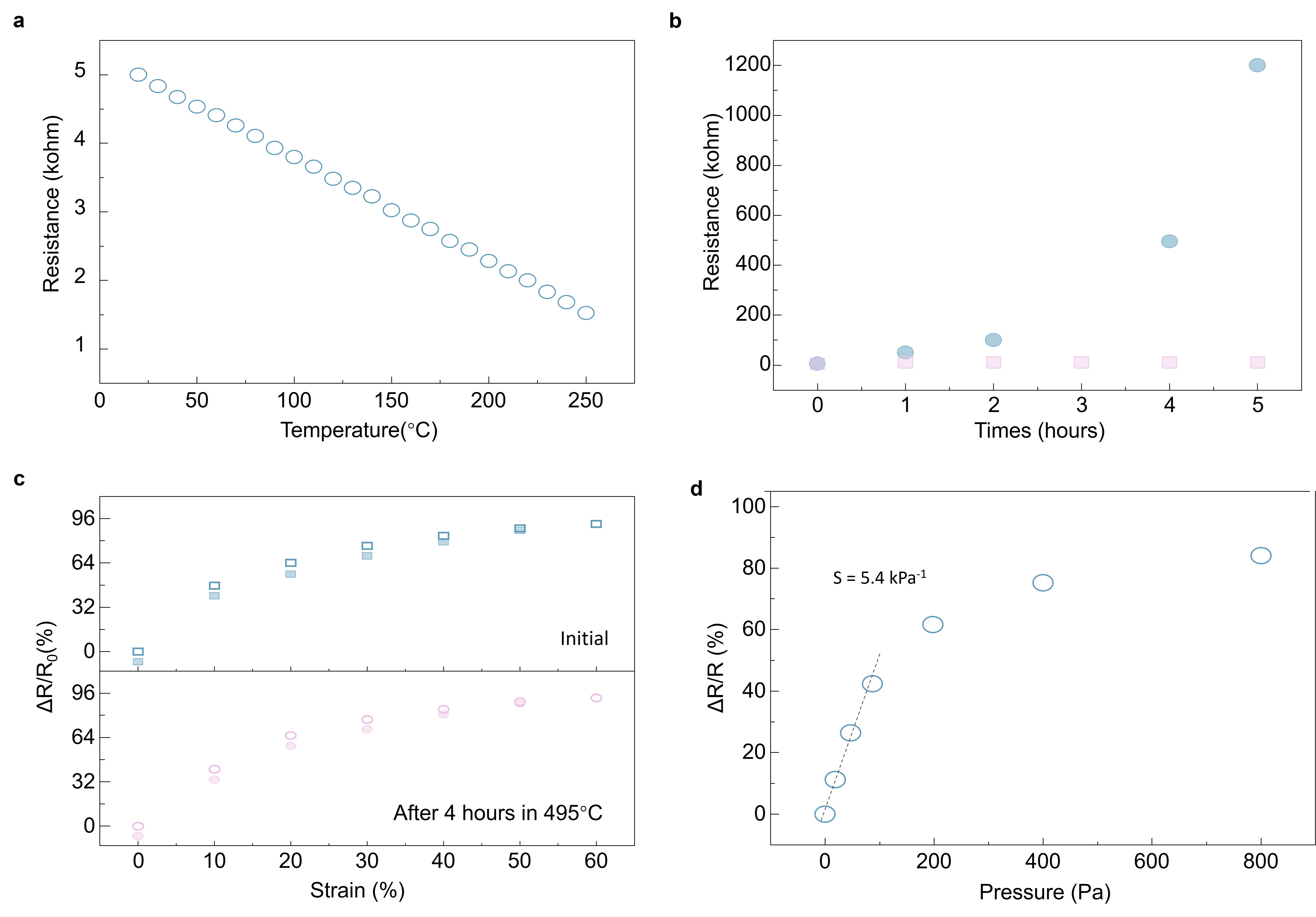
**

**Figure S5**. **Electrical Resistance Characteristics of the Piezoresistive Textile**. **a**, Electrical resistance as a function of temperature. **b**, Resistance measured at room temperature following prolonged exposure to various temperatures. **c**, Hysteresis curves illustrating piezoresistive performance at room temperature. **d**, Piezoresistive response as a function of pressure.

**
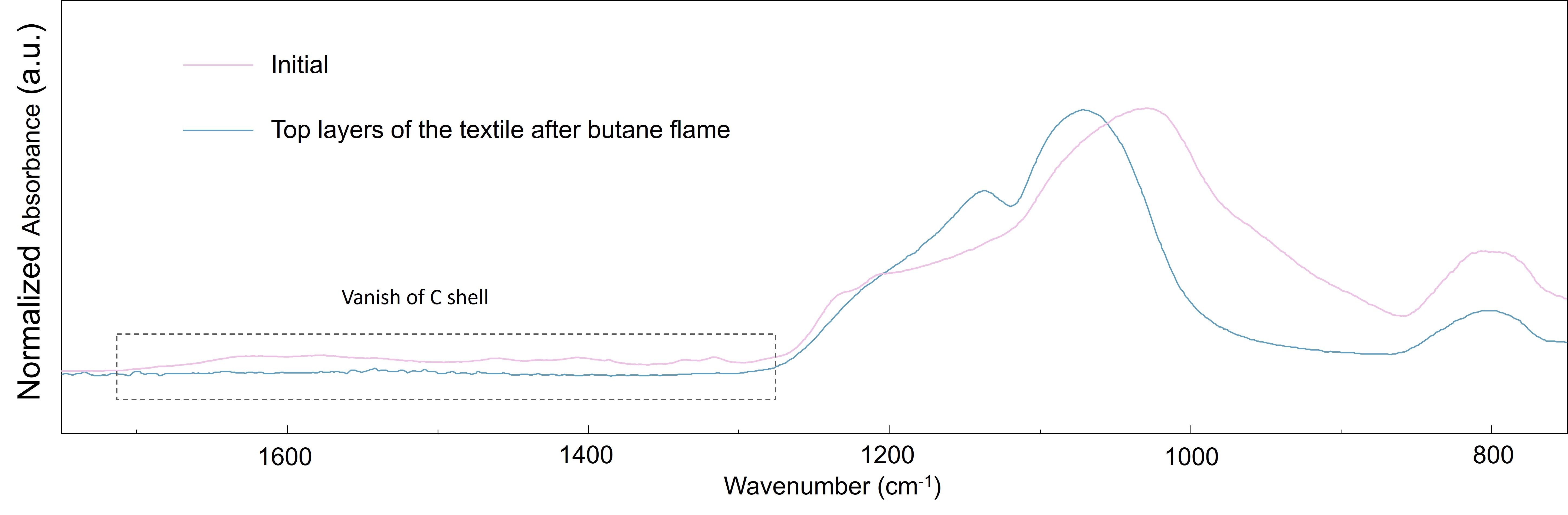
**

**Figure S6.** FTIR Spectrum of the Top Layers of the Textile After Exposure to the Butane Flame.


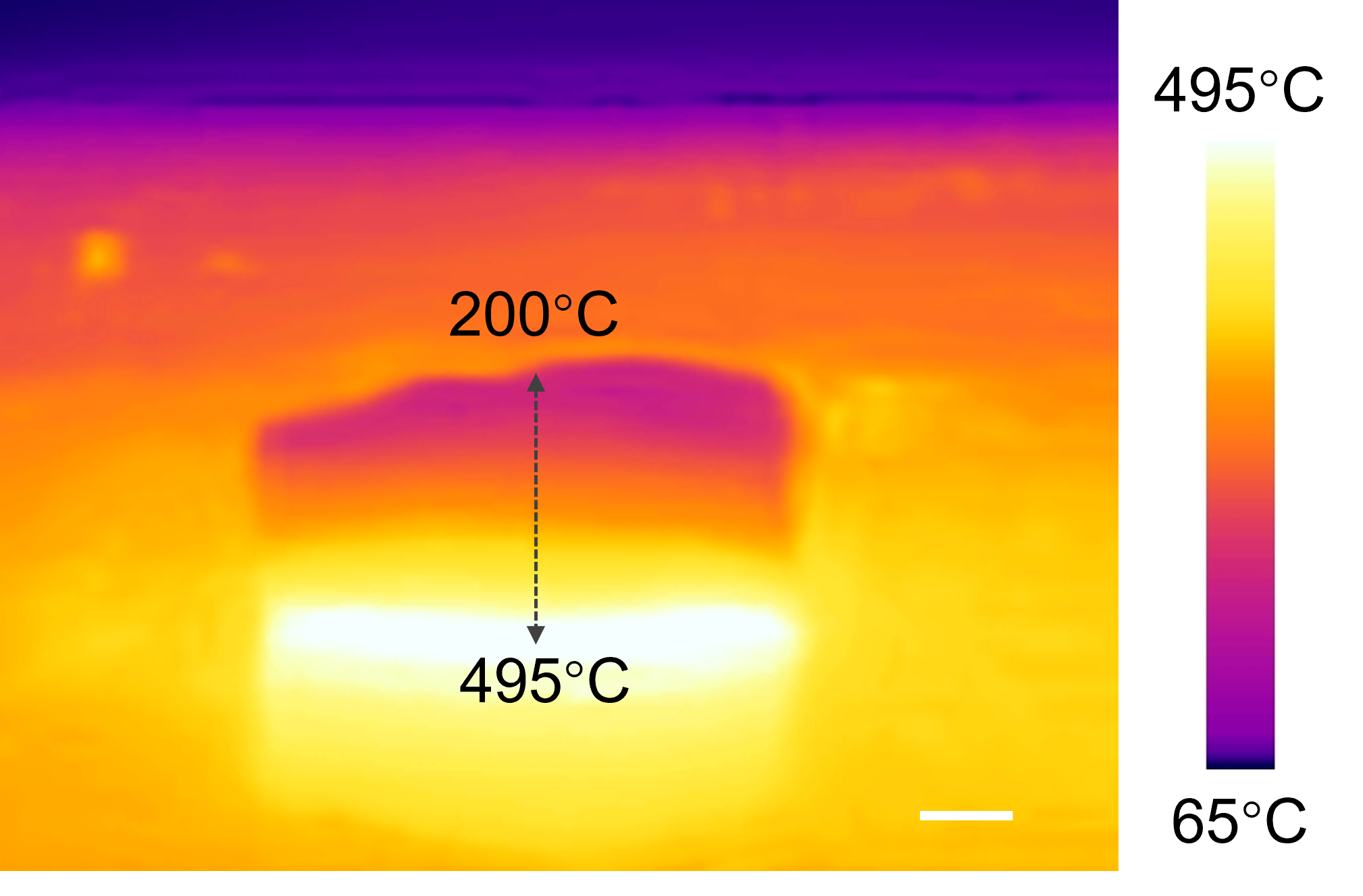


**Figure S7.** Infrared Image of a 3 mm Thick UTT Sensor on a 495°C Hot Plate (scale bar, 1 mm).

**
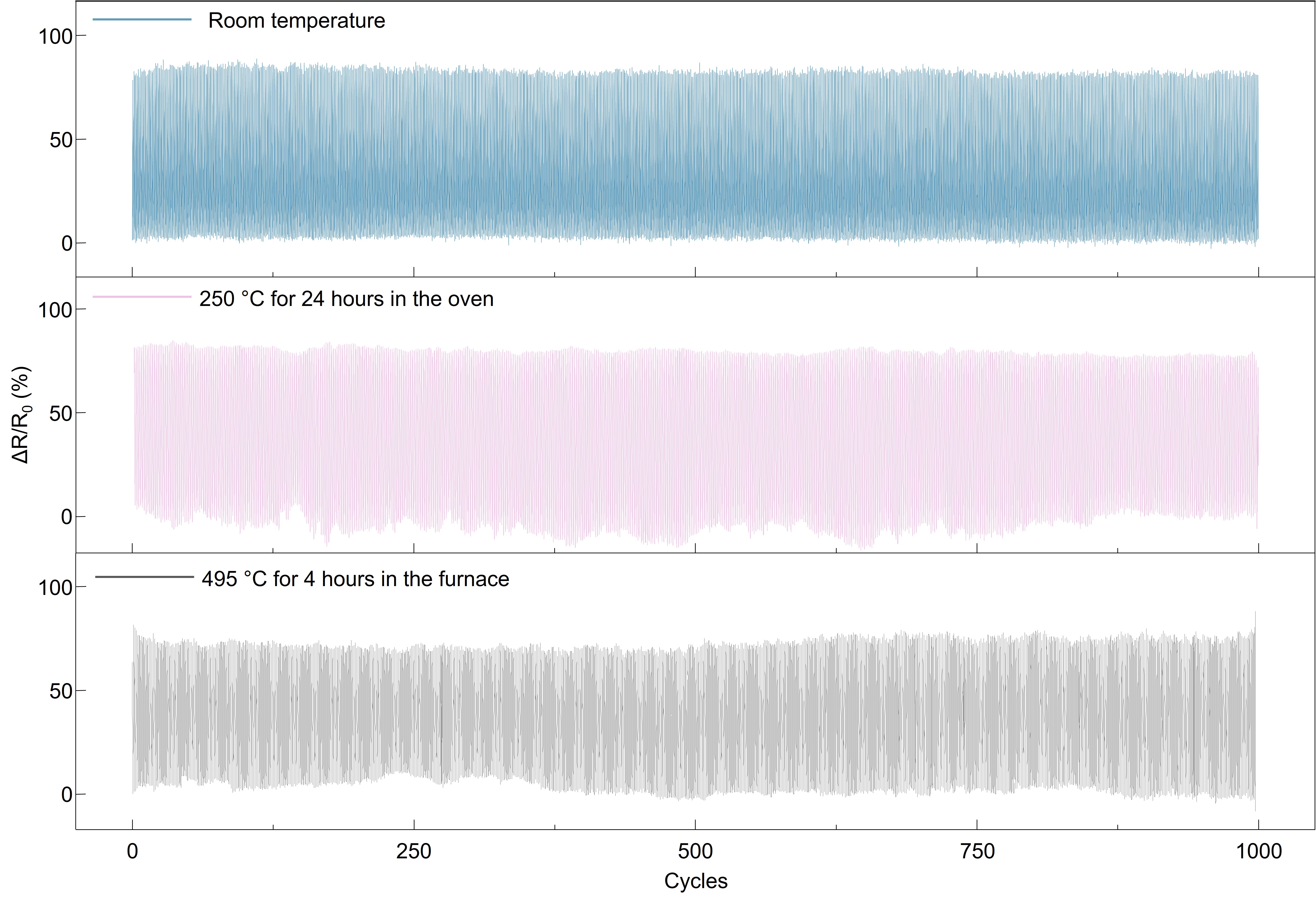
**

**Figure S8.** Durability test results for the sensor after exposure to 250°C in an oven for over 24 hours and results following exposure to 495°C in a furnace for 4 hours.

**
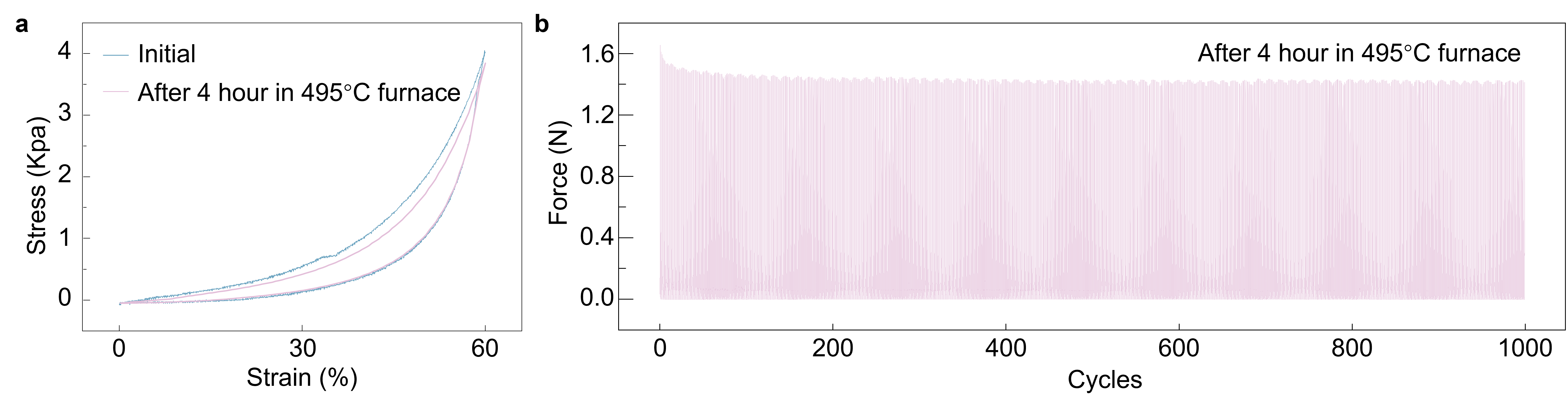
**

**Figure S9**. **Mechanical Resilience of the Textile**. **a**, Compressive stress-strain curves of the textile at initial state and after 4 hours in 495°C furnace. **b**, Durability test for up to 1,000 cycles at 60% strain.


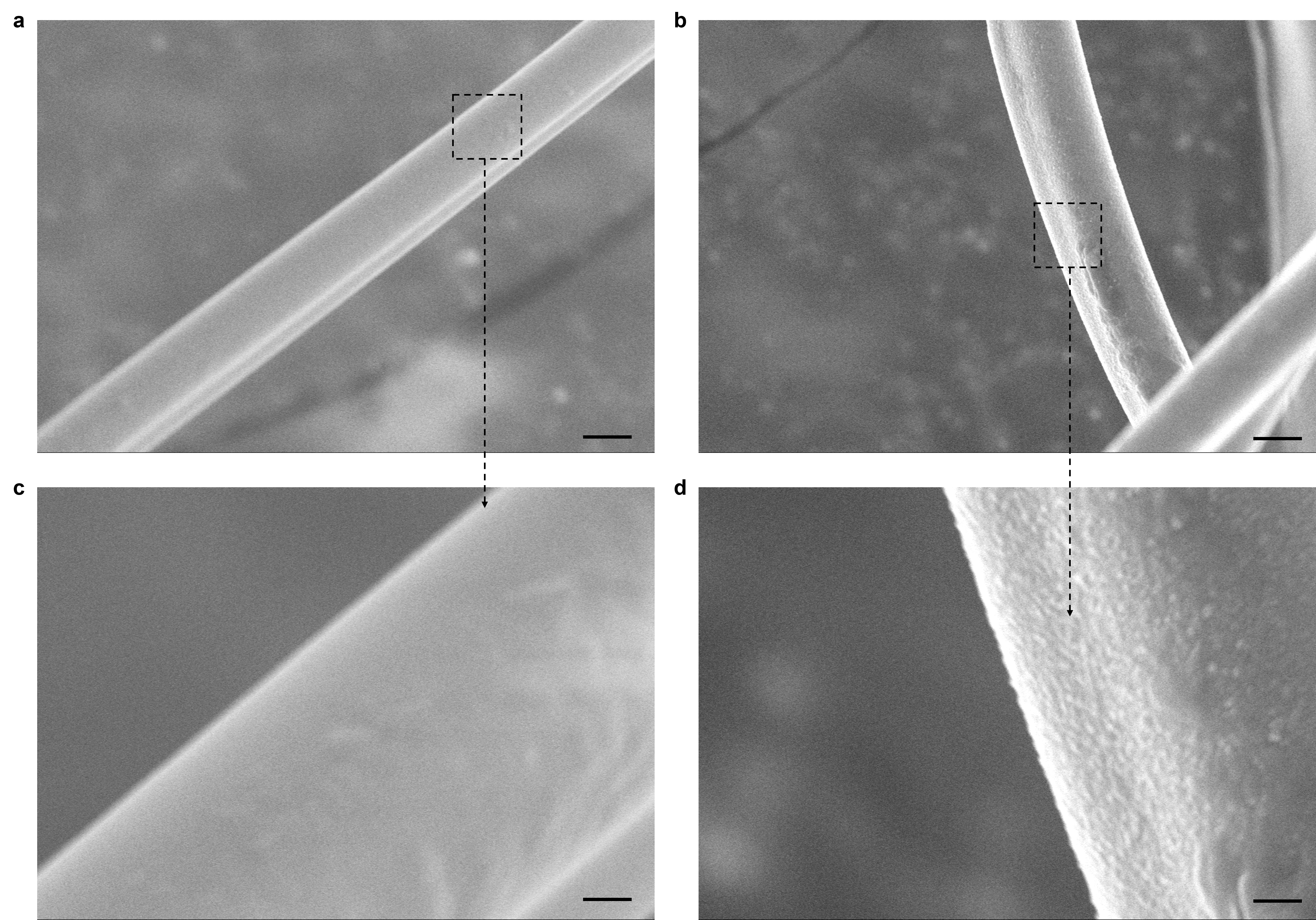


**Figure S10**. **Surface Morphology of Fiber for Failure Analysis**. **a**, Fiber surface in its initial state (scale bar, 1μm). **b**, Fiber surface after exposure to 495°C in a furnace for 5 hours (scale bar, 1μm). **c**, Zoomed-in image of the smooth initial surface (scale bar, 200 nm). **d**, Zoomed-in image of the roughened surface following thermal exposure (scale bar, 200 nm).


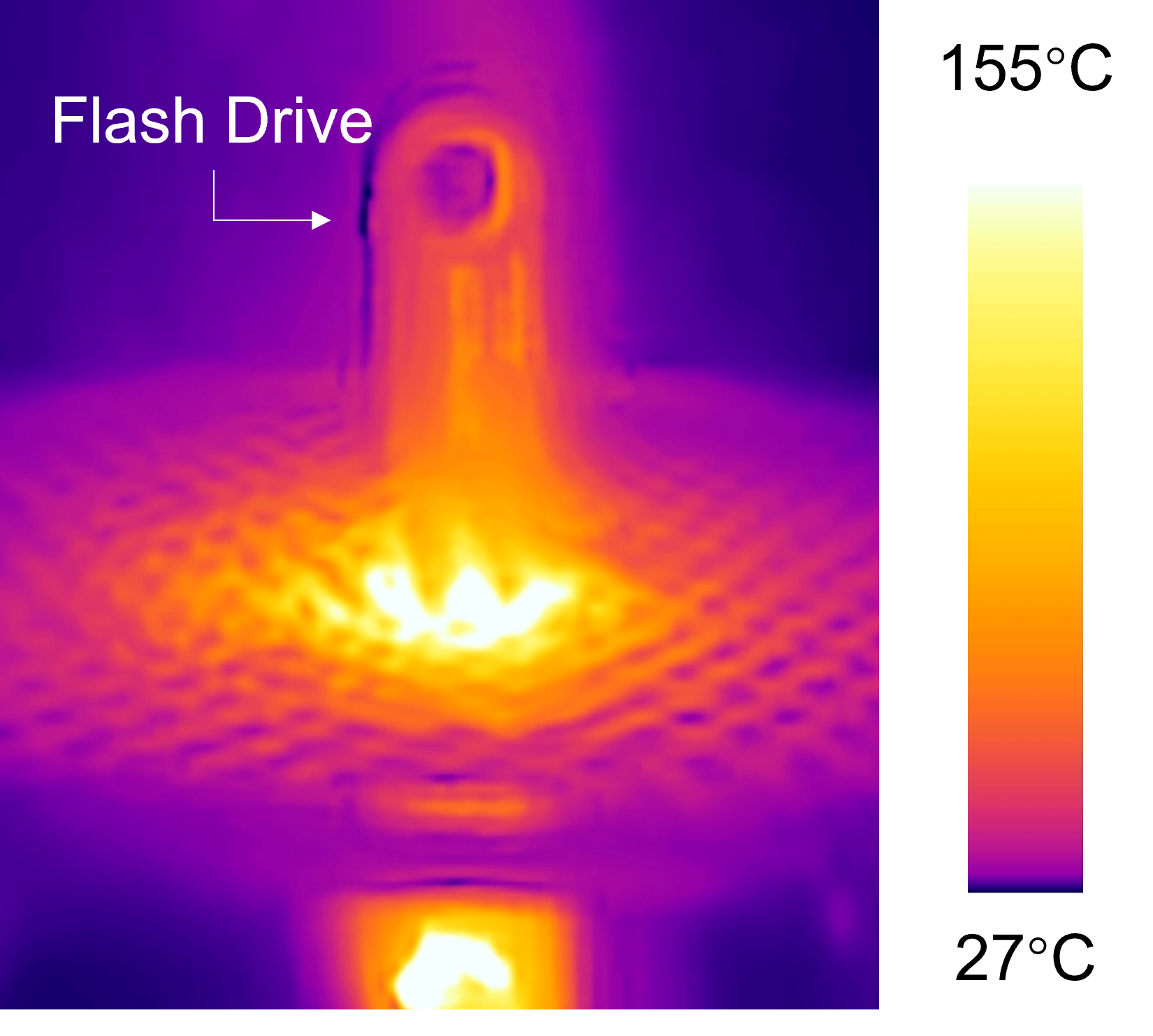


**Figure S11.** **Infrared Imaging of a USB Flash Drive in an Alcohol Flame for 15s.**

**Table S1:** Comparison of Thermal Stability with Other Flexible Piezoresistive Sensors.

| **Materials** | **Temperature (°C)** | **Duration** |
| --- | --- | --- |
| SiOC@C (This work) | 250 | > 24 hours |
| SiOC@C (This work) | 495 | 4 hours |
| MXene/Polyetherimide^[5]^ | 100 | Not reported, cycling stable |
| Au/Mxene/PDMS^[6]^ | 400 | Not reported, cycling stable |
| MXene/Polyetherimide^[7]^ | 200 | > 24 hours |

**Note S1: Permeability assessment** **of the piezoresistive textiles**

The piezoresistive textile presented in this work features core-shell fibers with an average diameter (*d*) of approximately 1.2 µm and a low bulk density of 58 mg/cm³ (0.058 g/cm³). This result indicates an exceptionally high porosity of approximately 97.4%, implying that the textile possesses an inherently open and breathable structure. Air permeability (*K*_air_) in nonwoven textiles is governed primarily by porosity and fiber dimensions. Using the Kozeny–Carman equation, a widely accepted theoretical approach for fibrous porous media, air permeability can be estimated as follows^[4]^:

$$K_{air}=\frac{\varepsilon^{3}}{{k\cdot S}^{2}\cdot\left( 1-\varepsilon\right)^{2}} (S1)$$

Where ε is porosity, S is specific surface aero per volume (4/d), k is Kozeny constant (4.5 for fiber mat). In our case, the permeability is about 2.46×10 ^−7^cm^2^, consistent with typical values for nonwoven membranes.

**Supporting References**

[1] F. Rubio, J. Rubio, J. L. Oteo, A FT-IR study of the hydrolysis of tetraethylorthosilicate (TEOS), *Spectrosc. Lett.* **1998**, *31* (1), 199. <https://doi.org/10.1080/00387019808006772>

[2] D. R. Baganizi, E. Nyairo, S. A. Duncan, S. R. Singh, V. A. Dennis, Interleukin-10 Conjugation to Carboxylated PVP-Coated Silver Nanoparticles for Improved Stability and Therapeutic Efficacy, *Nanomaterials* **2017**, *7* (7), 165. <https://doi.org/10.3390/nano7070165>

[3] I. A. Safo, M. Werheid, C. Dosche, M. Oezaslan,The role of polyvinylpyrrolidone (PVP) as a capping and structure-directing agent in the formation of Pt nanocubes, *Nanoscale Adv.* **2019**, *1* (8), 3095. <https://doi.org/10.1039/c9na00186g>

[4] P. Xu, B. Yu, Developing a new form of permeability and Kozeny–Carman constant for homogeneous porous media by means of fractal geometry, *Advances in Water Resources* **2008**, *31* (1), 74. <https://doi.org/https://doi.org/10.1016/j.advwatres.2007.06.003>

[5] Y. Xie, Y. Cheng, Y. Ma, J. Wang, J. Zou, H. Wu, Y. Yue, B. Li, Y. Gao, X. Zhang, C.-W. Nan, 3D MXene-Based Flexible Network for High-Performance Pressure Sensor with a Wide Temperature Range, *Advanced Science* **2023**, *10* (6), 2205303. <https://doi.org/https://doi.org/10.1002/advs.202205303>

[6] N. Yang, X. Yin, H. Liu, X. Zhou, X. Yan, Y. Zhao, T. Cheng, High-Performance Wearable Piezoresistive Sensor with a Wide Temperature Range via a Ti3C2Tx MXene/Au Dual-Layer Conductive Network and Microspike Structure, *ACS Applied Nano Materials* **2024**, *7* (14), 16964. <https://doi.org/10.1021/acsanm.4c03060>

[7] M. Jia, C. Yi, Y. Han, L. Wang, X. Li, G. Xu, K. He, N. Li, Y. Hou, Z. Wang, Y. Zhu, Y. Zhang, M. Hu, R. Sun, P. Tong, J. Yang, Y. Hu, Z. Wang, W. Li, W. Li, L. Wei, C. Yang, M. Chen, Hierarchical Network Enabled Flexible Textile Pressure Sensor with Ultrabroad Response Range and High-Temperature Resistance, *Advanced Science* **2022**, *9* (14), 2105738. <https://doi.org/https://doi.org/10.1002/advs.202105738>
